# Risk factors for renal impairment in primary aldosteronism: a retrospective study

**DOI:** 10.3389/fendo.2025.1606182

**Published:** 2025-08-11

**Authors:** Shuitao Qin, Jianling Li, Siying Wu, Sen Li, Jing Huang, Jie Yu, Chaoping Wei, Lixia Wei, Shuangbei Zhu, Shanshan Chen, Meilan Chen

**Affiliations:** Department of Hypertension, The First Affiliated Hospital of Guangxi Medical University, Nanning, Guangxi, China

**Keywords:** primary aldosteronism, renal damage, hypokalemia, 24-hour urinary potassium, hypertension

## Abstract

**Objective:**

This study aimed to investigate the risk factors associated with renal impairment among patients diagnosed with primary aldosteronism (PA).

**Methods:**

This study enrolled 147 PA patients who were initially classified into hypokalemic (n=56) and normokalemic (n=91) groups according to serum potassium levels, followed by subgroup stratification using combined adrenal venous sampling (AVS) and computed tomography (CT) diagnostic data. For comparison, 280 patients diagnosed with essential hypertension (EH) served as the control group. Data on general patient characteristics and biochemical markers from blood and urine samples were collected. The analysis involved comparing these indicators across groups and performing binary logistic regression to identify potential risk factors for renal damage.

**Results:**

When compared to the EH group, the PA group had lower serum potassium and heart rate (*P* < 0.05), but higher diabetes prevalence, standing plasma aldosterone concentration (PAC), serum sodium, albumin-to-creatinine ratio (ACR), and 24-hour urinary potassium excretion (*P* < 0.05). Among PA patients, the hypokalemic subgroup showed higher systolic/diastolic blood pressures, PAC, serum sodium, 24-hour urinary potassium, microalbumin, and ACR versus the normokalemic subgroup (*P* < 0.05). Compared with the IHA subgroup, the APA subgroup showed significantly higher standing PAC levels (*P* < 0.05). The classic APA subgroup exhibited elevated 24-hour urinary microprotein, ACR values, and hypokalemia prevalence relative to non-classic unilateral cases (*P* < 0.05). However, no significant differences emerged between unilateral and bilateral aldosterone secretion groups for serum potassium, PAC levels, or renal damage markers (*P*>0.05). Hypokalemia (*OR*=3.027) and urinary potassium (*OR*=1.052) predicted proteinuria (*P*<0.05).

**Conclusion:**

This study demonstrates that renal impairment is more pronounced in PA patients than in those with EH. Notably, the classic APA subtype exhibits particularly severe damage, specifically manifested by elevated urinary microalbumin excretion. Furthermore, concomitant hypokalemia in PA patients is associated with more severe renal impairment. Hypokalemia and increased 24-hour urinary potassium excretion emerge as key risk factors for renal damage within this patient population.

## 1 Introduction

Primary Aldosteronism (PA) represents a significant clinical syndrome predominantly characterized by hypertension, which may occur alongside hypokalemia. This condition arises from the adrenal cortex’s autonomous overproduction of aldosterone, leading to consequential effects including sodium retention, potassium excretion, increased blood volume, and diminished activity of the renin-angiotensin system (1, 2). As the foremost cause of secondary hypertension, PA’s recognition has been heightened through improved risk awareness and the implementation of standardized screening protocols, resulting in a notable increase in its detection rates. Recent epidemiological data from China have demonstrated that PA’s prevalence among individuals newly diagnosed with hypertension surpasses 4.0% (3). PA comprises six subtypes: APA, IHA, unilateral adrenal hyperplasia (UAH), familial hyperaldosteronism (FH), aldosterone-producing carcinoma, and ectopic aldosterone-secreting neoplasms (4). APA and IHA constitute the most prevalent forms, collectively accounting for approximately 95% of PA cases (4). Comparative analyses have further established that PA patients are at an elevated risk of renal target organ damage compared to those with Essential Hypertension (EH) (4–6). The extent of renal impairment in PA patients can vary significantly, influenced by factors such as the clinical subtype of PA, exposure to plasma aldosterone concentration (PAC), and the severity of electrolyte imbalances (7). Despite this knowledge, limited clinical literature exists on the differential clinical and renal damage characteristics between PA patients with or without hypokalemia. Furthermore, the phenotypic differences in clinical manifestations and renal injury patterns across etiological subtypes of primary aldosteronism (PA) remain understudied. This study seeks to delineate the clinical features and renal damage observed in PA patients, including subgroup analyses, to identify potential risk factors for renal impairment, enhance the understanding of PA’s clinical and renal damage profiles, and underscore the importance of early diagnosis, treatment, and prevention of renal damage in PA patients.

## 2 Methods

### 2.1 Participant recruitment

This investigation enrolled individuals admitted to the First Affiliated Hospital of Guangxi Medical University’s Hypertension Ward for hypertension evaluation between July 2018 and September 2020. Participants were required to cease all antihypertensive medications, including dihydropyridine calcium channel blockers (CCBs), angiotensin-converting enzyme inhibitors (ACEIs), angiotensin II receptor blockers (ARBs), and β-blockers, for a minimum of two weeks prior to admission, and diuretics for at least four weeks. Hypokalemic individuals had their potassium levels normalized through dietary adjustments to ensure standard sodium intake levels. Patients exhibiting uncontrolled blood pressure were administered α-receptor blockers (terazosin) and/or non-dihydropyridine calcium antagonists (verapamil) to maintain systolic blood pressure below 160mmHg (1mmHg=0.133kPa) (4). This study enrolled 147 PA patients, including 56 with hypokalemia and 91 normokalemic subjects, with subsequent subgroup stratification based on integrated adrenal venous sampling (AVS) and computed tomography (CT) diagnostic criteria. Additionally, 280 patients with essential hypertension (EH) diagnosed during the same period were included as controls.

#### 2.1.1 Inclusion criteria

Eligible participants for the PA group exhibited high clinical suspicion of PA, evidenced through measurements of standing plasma renin activity (PRA) or direct renin concentration (DRC), plasma aldosterone concentration (PAC), and the calculation of standing plasma aldosterone to renin ratio (ARR) at thresholds of ≥30 (ng/dl)/[ng/(ml·h)] or ≥57 (pg/ml)/(pg/ml) for PAC/PRA and PAC/DRC, respectively. Subjects exceeding these ARR thresholds underwent confirmatory tests such as the saline loading test or captopril suppression test.PA diagnosis was confirmed by either:

Post-saline infusion PAC >100 pg/ml (>10 ng/dL), or

Post-50mg-captopril reduction in PAC <30% or PAC >110 pg/ml (>11 ng/dL) (4).

#### 2.1.2 Exclusion criteria

Exclusion criteria encompassed individuals with secondary hypertension types other than PA, determined through historical, physical, laboratory, and auxiliary examinations; those with severe liver, kidney, heart dysfunction, and malignant tumors; recent trauma, surgery, infection; and special conditions including pregnancy and lactation.

### 2.2 Analytical methods

#### 2.2.1 Data collection

Collected data included systolic blood pressure (SBP), diastolic blood pressure (DBP), heart rate(HR), gender, age, hypertension duration, height, weight, and body mass index (BMI), calculated as weight (kg) divided by height squared (m^2^).

#### 2.2.2 Biochemical analysis

Biochemical parameters were assessed using the Hitachi 7600 automatic analyzer, encompassing triglycerides (TG), cholesterol (TC), high-density lipoprotein cholesterol (HDL-C), low-density lipoprotein cholesterol (LDL-C), potassium, sodium, uric acid (UA), urea, creatinine (Cr), fasting plasma glucose (FPG), postprandial 2-hour blood glucose, glycated hemoglobin (HbA1C), urinary microalbumin to creatinine ratio (ACR), 24-hour urinary microprotein, potassium, and sodium. The normative upper limit for low potassium was set between 3.5 to 3.8 mmol/L, with levels below 3.5 mmol/L classified as hypokalemia (8). The estimated glomerular filtration rate (eGFR) was calculated utilizing the modified MDRD equation adapted for the Chinese population (9).

#### 2.2.3 Renal damage diagnostic criteria

(10): categorizing eGFR <60 ml/(min·1.73m^2^) as a decrease in eGFR,ACR≥30mg/g or 24-hour urinary microalbumin ≥30mg/24h was defined as proteinuria.

#### 2.2.4 Assessment of lipid and glucose metabolism disorders

Lipid abnormalities were diagnosed based on 2016 “Chinese Guidelines for the Prevention and Treatment of Dyslipidemia in Adults” (11), and type 2 diabetes was determined according to the “Chinese Guidelines for the Prevention and Treatment of Type 2 Diabetes (2022 Edition)”, with specific criteria detailed for each condition (12).

#### 2.2.5 Hormone quantification

Subjects maintained a non-recumbent position for at least two hours upon awakening, followed by a 5–15 minute rest before blood sampling for standing PRA or DRC and PAC measurement and ARR calculation, conducted in the hospital’s Nuclear Medicine Department using radioimmunoassay (RIA) and chemiluminescence immune assay (CLIA) methodologies as appropriate.

#### 2.2.6 Adrenal CT and AVS

All diagnosed primary aldosteronism (PA) patients underwent:

**Adrenal CT:** 64-slice thin-section contrast-enhanced CT (Radiology Department).

Normal anatomy: “Y” or inverted “V” configuration on axial view (13).

Adenoma criteria (14):

1. Unenhanced CT value <10 HU (calcifications and hemorrhagic foci must be excluded during measurement).
2. If CT value >10 HU, further contrast-enhanced CT washout analysis is required: Absolute washout ≥60% or relative washout ≥40% supports adenoma diagnosis.

**AVS:** all procedures were conducted before 12:00 noon without ACTH stimulation (Catheterization Laboratory).

1. Selectivity Index (SI) = Adrenal vein cortisol/Peripheral vein cortisol.

Successful cannulation: SI ≥2.

2. Lateralization Index (LI) = (Aldosterone/cortosterone in dominant adrenal vein)/(Contralateral aldosterone/cortisol).

Unilateral hypersecretion: LI ≥4 (15).

Based on adrenal CT and AVS results, PA patients were further stratified into APA and IHA subgroups (4).

APA subgroup: Defined by concurrent fulfillment of:

1. Adrenal CT demonstrating adenoma criteria.
2. AVS-confirmed unilateral aldosterone hypersecretion.

IHA subgroup: Defined by either:

1. Normal or bilaterally hyperplastic adrenals on CT.
2. AVS evidence of bilateral aldosterone secretion.

Based on the combined results of CT and AVS, we further subdivided the unilateral secretion group into two the classical APA subgroup and the nonclassical secretion subgroup (16):

Classical APA Group (CT-adenoma phenotype): Requires concurrent fulfillment of (irrespective of CYP11B2 staining):

1. Imaging criterion: Presence of a solitary adrenal nodule (1–4 cm) with adenoma features;
2. Functional criterion: AVS-confirmed unilateral aldosterone excess ipsilateral to the identified nodule.

Non-classical Group: Requires concurrent fulfillment of:

1. Imaging criterion: Normal adrenals/bilateral hyperplasia or multiple nodules (including unilateral multiple nodules).
2. Functional criterion: AVS-confirmed unilateral aldosterone excess.

#### 2.2.7 Statistical analysis

Employed SPSS 22.0 for data analysis, expressing normally or approximately normally distributed quantitative data as mean ± standard deviation, and utilizing independent sample t-tests or non-parametric tests for intergroup comparisons. Categorical data were analyzed via the χ2 test, with logistic regression applied to discern risk factors for renal impairment in PA patients, considering *P* < 0.05 as statistically significant.

## 3 Results

### 3.1 Comparative analysis between PA and EH groups

#### 3.1.1 Demographic and clinical characteristics

In comparison with the EH group, the PA group demonstrated significantly lower levels of serum potassium and HR, alongside higher proportions of diabetes comorbidity, standing PAC, serum sodium and 24-hour urinary potassium (all *P*<0.05). There were no statistically significant differences between the groups regarding gender, age, disease duration, BMI, SBP, DBP, UA, HbA1C, TG, TC, LDL-C, HDL-C, lipid abnormalities, FPG, and postprandial 2-hour blood glucose levels (*P*>0.05) (**Table 1**).

**Table 1:** Clinical characteristics of study participants.

Clinical characteristics	PA (n=147)	EH (n=280)	*P*
Age (y)	46.14 ± 10.51	44.57 ± 13.39	0.182
Duration of hypertension (month)	47.78 ± 56.28	45.60 ± 61.84	0.721
Sex (male/woman) (n)	67/80	147/133	0.154
BMI (kg/m^2^)	24.15 ± 3.39	24.23 ± 3.17	0.054
SBP (mmHg)	149.65 ± 28.67	151.51 ± 19.51	0.432
DBP (mmHg)	89.92 ± 19.45	92.07 ± 15.66	0.22
HR (times/min)	78.43 ± 16.29	84.03 ± 13.81	<0.001
Serum potassium (mmol/L)	3.56 ± 0.62	3.87 ± 0.34	<0.001
Hypokalemia[n (%)]	56 (38.00%)	33 (11.70%)	<0.001
Serum sodium (mmol/L)	141.33 ± 2.06	140.74 ± 2.27	0.009
Urea (mmol/L)	4.73 ± 1.40	4.85 ± 1.34	0.418
UA (umol/L)	327.99 ± 106.66	339.97 ± 100.30	0.058
TG (mmol/L)	1.83 ± 2.03	1.80 ± 3.89	0.918
TC (mmol/L)	4.85 ± 0.99	4.89 ± 0.97	0.709
HDL-C (mmol/L)	1.20 ± 0.31	1.24 ± 0.32	0.189
LDL-C (mmol/L)	2.86 ± 0.87	2.96 ± 0.84	0.253
Lipid abnormalities[n (%)]	122 (43.57%)	59 (40.13%)	0.534
FBG (mmol/L)	4.77 ± 0.97	4.74 ± 0.72	0.720
Postprandial 2-hour blood glucose (mmol/L)	6.52 ± 2.65	6.46 ± 2.09	0.818
HbA1C (%)	5.53 ± 0.98	5.59 ± 0.78	0.055
Combined diabetes[n (%)]	21 (14.29%)	14 (5.00%)	0.001
Standing PAC (pg/ml)	245.76 ± 192.22	199.20 ± 47.70	0.005
24-hour urinary potassium (mmol/24h)	36.41 ± 18.56	28.94 ± 12.30	0.041
24-hours urinary sodium (mmol/24h)	102.65 ± 45.08	106.25 ± 30.26	0.245

PA, primary aldosteronism; EH, essential hypertension; BMI, body mass index; SBP, systolic blood pressure; DBP, Diastolic blood pressure; HR, heart rate; UA, uric acid; TG, triglyceride; TC, cholesterol; HDL-C, high-density lipoprotein cholesterol; LDL-C, low-density lipoprotein cholesterol; FBG, fasting plasma glucose; HbA1C, glycated haemoglobin; PAC, plasma aldosterone concentration.

#### 3.1.2 Renal damage indicators

The PA group exhibited a higher ACR compared to the EH group (P<0.05). However, Cr, eGFR, and 24-hour urinary microalbumin did not differ significantly between the groups (*P*>0.05) (**Table 2**).

**Table 2:** Renal damage indicators of study participants.

Renal damage indicators	PA (n=147)	EH (n=280)	*P*
Cr (umol/L)	75.50 ± 24.55	76.52 ± 18.79	0.657
eGFR[ ml/min/1.73 m2]	102.49 ± 27.03	101.59 ± 23.29	0.719
24-hour urinary microprotein (mg/24h)	13.8 (6.95,27.45)	10.10 (4.88,22.50)	0.092
ACR (mg/g)	9.56 (5.08,22.1)	7.07 (3.54,15.91)	0.034

PA, primary aldosteronism; EH, essential hypertension; Cr, creatinine; eGFR, estimated glomerular filtration rate; ACR, Albumin-to-Creatinine Ratio.

### 3.2 Hypokalemia *vs* normokalemia subgroups within the PA cohort

#### 3.2.1 General data

The hypokalemia subgroup showed significantly higher SBP, DBP, standing PAC, serum sodium, and 24-hour urinary potassium compared to the normokalemia subgroup (*P*<0.05). No significant differences were observed in age, disease duration, gender, BMI, HR, urea, UA, TG, TC, HDL-C, LDL-C, lipid abnormalities, FPG, postprandial 2-hour glucose, HbA1C, diabetes comorbidity, and 24-hour urinary sodium (*P*>0.05) (**Table 3**).

**Table 3:** Clinical characteristics of subgroups within the PA cohort.

Clinical characteristics	The hypokalemia subgroup (n=56)	The normokalemia subgroup (n=91)	*P*
Age (y)	44.95 ± 10.73	46.61 ± 10.16	0.348
Duration of hypertension (month)	50.34 ± 53.10	47.14 ± 58.66	0.742
Sex (male/woman) (n)	31/26	37/54	0.103
BMI (kg/m^2^)	23.84 ± 4.16	23.32 ± 2.85	0.456
SBP (mmHg)	156.72 ± 18.56	150.22 ± 18.96	0.047
DBP (mmHg)	95.57 ± 15.31	89.45 ± 13.81	0.015
HR (times/min)	80.22 ± 11.02	79.92 ± 12.38	0.884
Serum potassium (mmol/L)	3.02 ± 0.31	3.94 ± 0.32	<0.001
Serum sodium (mmol/L)	141.92 ± 2.12	140.98 ± 1.96	0.008
Urea (mmol/L)	4.60 ± 1.28	4.88 ± 1.38	0.24
UA (umol/L)	314.30 ± 95.99	340.56 ± 107.21	0.141
TG (mmol/L)	1.45 ± 1.26	2.04 ± 2.36	0.052
TC (mmol/L)	4.63 ± 0.82	4.93 ± 1.18	0.071
HDL-C (mmol/L)	1.18 ± 0.28	1.20 ± 0.35	0.651
LDL-C (mmol/L)	2.75 ± 0.69	2.90 ± 1.00	0.318
Lipid abnormalities[n (%)]	18 (32.14%)	41 (45.05%)	0.121
FBG (mmol/L)	4.66 ± 0.90	4.84 ± 1.00	0.258
Postprandial 2-hour blood glucose (mmol/L)	6.38 ± 1.97	6.75 ± 2.86	0.400
HbA1C (%)	5.44 ± 0.747	5.64 ± 0.927	0.181
Combined diabetes[n (%)]	7 (12.5%)	14 (15.38%)	0.627
StandingPAC (pg/ml)	298.65 ± 300.826	211.10 ± 50.65	0.037
24-hour urinary potassium (mmol/24h)	40.40 ± 21.78	32.94 ± 14.27	0.039
24-hours urinary sodium (mmol/24h)	102.65 ± 46.08	109.25 ± 70.25	0.565

BMI, body mass index; SBP, systolic blood pressure; DBP, Diastolic blood pressure; HR, heart rate; UA, uric acid; TG, triglyceride; TC, cholesterol; HDL-C, high-density lipoprotein cholesterol; LDL-C, low-density lipoprotein cholesterol; FBG, fasting plasma glucose; HbA1C, glycated haemoglobin; PAC, plasma aldosterone concentration.

#### 3.2.2 Renal damage indicators

The hypokalemia subgroup demonstrated significantly higher levels of 24-hour urinary microalbumin and ACR compared to the normokalemia subgroup (*P*<0.01). Differences in Cr and eGFR were not statistically significant (*P*>0.05) (**Table 4**).

**Table 4:** Renal damage indicators of subgroups within the PA cohort.

Renal damage indicators	The hypokalemia subgroup (n=56)	The normokalemia subgroup (n=91)	*P*
Cr (umol/L)	75.96 ± 20.191	76.04 ± 25.88	0.984
eGFR[ ml/min/1.73 m2]	103.70 ± 26.79	101.76 ± 27.18	0.776
24-hour urinary microprotein (mg/24h)	19.10 (12.00,34.50)	10.15 (5.02,20.79)	<0.001
ACR (mg/g)	15.91 (8.84,46.85)	8.84 (3.54,21.22)	<0.001

Cr, creatinine; eGFR, estimated glomerular filtration rate; ACR, Albumin-to-Creatinine Ratio.

### 3.3 Comparative analysis the unilateral secretion subgroup and the bilateral secretion subgroup

No significant differences were observed between the two groups in serum potassium levels, hypokalemia incidence, 24-hour urinary potassium excretion, PAC, or renal injury markers—including 24-hour urinary microalbumin, ACR, Cr, and eGFR (*P*>0.05) (**Table 5**).

**Table 5:** Comparison between the unilateral secretion subgroup and the bilateral secretion subgroup.

Clinical characteristics	Unilateral (n=46)	Bilateral (n=101)	*P*
Cr (umol/L)	76.00 ± 28.59	76.31 ± 22.21	0.349
eGFR[ ml/min/1.73 m2]	100.72 ± 27.38	103.00 ± 26.87	0.915
24-hour urinary microprotein (mg/24h)	15.91 (7.92,29.98)	13.97 (5.92,26.92)	0.156
ACR (mg/g)	9.99 (5.98,28.02)	9.10 (4.04,18.58)	0.368
Standing PAC (pg/ml)	276.85 ± 305.40	235.49 ± 138.38	0.096
Serum potassium (mmol/L)	3.58 ± 0.64	3.59 ± 0.51	0.171
Hypokalemia[n (%)]	19 (42%)	37 (36.63)	0.691
24-hour urinary potassium (mmol/24h)	34.31 ± 14.36	36.47 ± 18.92	0.157

Cr, creatinine; eGFR, estimated glomerular filtration rate; ACR, Albumin-to-Creatinine Ratio; PAC, plasma aldosterone concentration.

### 3.4 Comparative analysis between the APA subgroup and the IHA subgroup

Compared with the IHA subgroup, the APA subgroup demonstrated significantly higher Standing PAC levels (P<0.05). However, no statistically significant differences were observed between the groups in serum potassium levels, hypokalemia incidence, 24-hour urinary potassium excretion, or renal injury markers—including 24-hour urinary microalbumin excretion, ACR, Cr, and eGFR (*P*>0.05) (**Table 6**).

**Table 6:** Comparison between the APA subgroup and the IHA subgroup.

Clinical characteristics	APA (n=27)	IHA (n=101)	*P*
Cr (umol/L)	80.31 ± 27.42	76.42 ± 22.62	0.580
eGFR[ ml/min/1.73 m2]	95.63 ± 25.40	102.93 ± 26.93	0.690
24-hour urinary microprotein (mg/24h)	16.31 (8.20,28.50)	13.12 (5.21,27.56)	0.110
ACR (mg/g)	11.02 (4.96,26.95)	8.98 (8,10,20.70)	0.301
Standing PAC (pg/ml)	321.62 ± 422.077	236.51 ± 140.21	0.012
Serum potassium (mmol/L)	3.28 ± 0.60	3.52 ± 0.51	0.520
Hypokalemia[n (%)]	15 (55.56%)	37 (36.66%)	0.083
24-hours urinary sodium (mmol/24h)	34.65 ± 16.47	36.43 ± 19.11	0.513

Cr, creatinine; eGFR, estimated glomerular filtration rate; ACR, Albumin-to-Creatinine Ratio; PAC, plasma aldosterone concentration.

### 3.5 Comparative analysis between the classic APA subgroup and the non-classic subgroup

Compared to the non-classic unilateral subgroup, the classic APA subgroup exhibited significantly higher values of 24-hour urinary microprotein, ACR, and hypokalemia prevalence (p < 0.05). However, no significant intergroup differences were observed in serum potassium, 24-hour urinary potassium, standing PAC, eGFR, or Cr (*P*>0.05) (**Table 7**).

**Table 7:** Comparative between the classic APA subgroup and the non-classic unilateral subgroup.

Clinical characteristics	Classic APA (n=27)	Non-classic unilateral (n=19)	*P*
Cr (umol/L)	80.11 ± 27.52	71.65 ± 29.90	0.938
eGFR[ ml/min/1.73 m2]	95.13 ± 24.80	106.66 ± 30.50	0.612
24-hour urinary microprotein (mg/24h)	16.87 (9.91,32.16)	12.10 (4.78,27.79)	0.027
ACR (mg/g)	11.21 (7.01,25.31)	7.73 (5.84,16.62)	0.017
Standing PAC (pg/ml)	321.41 ± 421.64	229.66 ± 68.66	0.122
Serum potassium (mmol/L)	3.28 ± 0.60	3.89 ± 0.54	0.072
Hypokalemia[n (%)]	15 (55.56%)	4 (21.05%)	0.028
24-hour urinary potassium (mmol/24h)	34.65 ± 16.47	33.83 ± 11.39	0.311

Cr, creatinine; eGFR, estimated glomerular filtration rate; ACR, Albumin-to-Creatinine Ratio; PAC, plasma aldosterone concentration.

### 3.6 Proteinuria-related factor analysis within the PA group

A univariate logistic regression analysis was conducted with the presence of proteinuria (ACR≥30mg/g or 24-hour urinary microalbumin ≥30mg/24h) as the dependent variable. Independent variables included demographic and clinical parameters such as gender, age, hypertension duration, BMI, SBP, DBP, HR, hypokalemia, serum sodium, urea, Cr, UA, TG, TC, HDL-C, LDL-C, lipid abnormalities, FBG, postprandial 2-hour glucose, HbA1C, diabetes comorbidity, standing PAC, and 24-hour urinary potassium and sodium. The analysis revealed positive correlations of SBP, DBP, HR, hypokalemia, FBG, 24-hour urinary potassium, and sodium with proteinuria, while age showed a negative correlation (*P*<0.05). A multivariate binary logistic regression model, incorporating variables with *P*<0.05, identified hypokalemia (*OR*=3.027) and 24-hour urinary potassium (*OR*=1.052) as independent predictors of proteinuria in PA patients (*P*<0.05) (**Table 8**).

**Table 8:** Multivariate binary logistic regression analysis of proteinuria-related factors within the PA group.

Relevant factor	*B*	*SE*	*X2*	*P*	*OR*	95%*CI*
hypokalemia^a^	1.108	0.486	5.184	0.023	3.027	1.167∼7.854
24-hour urinary potassium(mmol/24h)^b^	0.05	0.015	10.74	0.001	1.052	1.020∼1.084

The independent variables included in regression model a were age, SBP, DBP, HR, hypokalemia, FBG, and 24h sodium; The independent variables included in the regression model b were age, SBP, DBP, HR, 24h urine potassium, FBG, 24h urine sodium; PA, primary aldosteronism.

### 3.7 Factors associated with reduced eGFR within the PA group

Using reduced eGFR as the dependent variable, a univariate logistic regression analysis with variables excluding gender, age, and Cr due to the modified MDRD equation’s adjustments showed positive correlations with the duration of hypertension, SBP, DBP, urea, UA, TG, FBG, and postprandial 2-hour glucose. In contrast, standing PAC, HDL-C, and LDL-C showed negative correlations (*P*<0.05). Further multivariate analysis did not identify these factors as independent predictors of reduced eGFR in PA patients (*P*>0.05).

### 3.8 Analysis of proteinuria and reduced eGFR factors within the hypokalemia subgroup of PA

The analysis of the hypokalemia duration and its relation to proteinuria and reduced eGFR within the hypokalemia subgroup did not show significant correlations (*P*>0.05).

### 3.9 Analysis of proteinuria and reduced eGFR within the EH and the subgroups of PA

No significant associations were observed between hypertension duration and proteinuria or reduced eGFR in either the EH group or the Subgroups of PA(APA *vs* IHA; Hypokalemic *vs* normokalemic; Unilateral *vs* bilateral secretion) (all *P* > 0.05).

### 3.10 Association between hypokalemia and disease duration in PA patients

No significant association was found between the duration of hypertension (disease duration) and the occurrence of hypokalemia in PA patients (*P* > 0.05).

## 4 Discussion

Primary Aldosteronism (PA) is a leading cause of secondary hypertension and presents a notable health concern due to its association with an increased risk of hypertension-induced renal damage compared to Essential Hypertension (EH) (17–19). This elevated risk emphasizes the importance of identifying clinical characteristics and understanding renal damage in PA patients to enable early diagnosis and timely intervention. Such measures are crucial for reducing the risk of renal complications and improving long-term patient outcomes.

Clinically, PA manifests through several distinct markers: elevated plasma aldosterone concentration (PAC), suppressed plasma renin activity, hypertension, and a dysregulated sodium and potassium balance (20). The presence of hypokalemia in PA patients, while variable, is particularly concerning as it not only indicates altered potassium homeostasis but also serves as a significant risk factor for renal impairment. Despite the variability in hypokalemia among PA patients, the condition necessitates vigilant screening for PA among all hypertensive individuals, irrespective of their potassium levels (21, 22).

Patients with PA often have elevated PAC, which significantly affects renal ion transporters and potassium homeostasis, especially key ion transporters for sodium and potassium handling, such as the epithelial sodium channel (ENaC) and the renal outer medullary potassium channel (ROMK). Studies have shown that PAC regulates the activity of ENaC through various mechanisms, thereby affecting sodium reabsorption (23). Specifically, PAC can increase the expression and activity of ENaC, mainly by promoting the membrane insertion of ENaC subunits and reducing their degradation (24).For example, PAC can activate protein kinase B (Akt) and serum glucocorticoid kinase 1 (SGK1), inhibit the activity of Nedd4-2, and thus reduce the ubiquitination and degradation of ENaC. This regulatory effect is crucial for maintaining sodium balance and blood pressure stability (25).

The ROMK channel plays a key role in renal potassium secretion. Elevated PAC levels can affect the activity of ROMK through various pathways. On the one hand, aldosterone can increase the expression of ROMK, promoting potassium secretion; on the other hand, aldosterone can also indirectly affect the function of ROMK by regulating the activity of other ion transporters (such as the sodium-chloride cotransporter NCC). This regulatory mechanism helps maintain potassium homeostasis, but in PA patients, the high PAC level may lead to excessive potassium secretion, thereby causing hypokalemia (26).

Moreover, PA patients have chronic elevated PAC, which has a profound impact on cellular processes, especially through various mechanisms triggering inflammation, oxidative stress, and fibrosis (27).These cellular processes not only affect the kidneys but also involve multiple organs such as the heart and blood vessels, leading to severe tissue damage and functional disorders. These mechanisms involve not only the activation of the mineralocorticoid receptor by aldosterone but also its regulation of oxidative stress and inflammatory signaling pathways (28). Therefore, interventions targeting aldosterone and its downstream signaling pathways, such as aldosterone synthase inhibitors and mineralocorticoid receptor antagonists, are expected to become effective strategies for treating PA-related organ damage (29).

Recent studies have reinforced the link between PA and renal damage (30–32). The results of this study showed elevated urinary microalbumin excretion in PA patients, especially those with hypokalemia, highlight the severity of renal impairment associated with this condition. Studies have shown that in the early stages of primary aldosteronism (PA), the degree of renal function impairment may be masked by glomerular hyperfiltration. Elevated plasma aldosterone concentration (PAC) helps maintain the glomerular filtration rate, making early renal damage less detectable (33). In fact, the increase in urinary microalbumin is a sensitive indicator of early renal damage (34). In this study, all PA patients included were in the early stage of the disease, thus only showing increased urinary microalbumin excretion, while the estimated glomerular filtration rate (eGFR) and serum creatinine levels remained within normal ranges.

Hypokalemia leads to renal damage through various mechanisms, including renal tubular dysfunction, reduced renal blood flow and GFR, changes in renal morphology, inflammation and fibrosis, as well as oxidative stress. These mechanisms interact with each other, ultimately resulting in progressive renal dysfunction (35). These indicators are corroborated by a growing body of literature (36–39) that identifies hypokalemia and 24-hour urinary potassium excretion as significant predictors of renal damage in these patients. For instance, research by Rossi et al (40, 41) demonstrated that hypokalemia in PA is strongly linked with increased renal fibrosis and reduced glomerular filtration rate, underscoring the critical impact of electrolyte disturbances on renal health in these individuals.

PA is predominantly classified into APA and IHA, with APA patients typically exhibiting unilateral adrenal adenomas and more severe renal injury (29)—a pathology directly linked to hyperaldosteronism-induced tubulointerstitial fibrosis, glomerulosclerosis, and mineralocorticoid receptor (MR)-mediated dysregulation of tubular sodium-potassium exchange, culminating in hypokalemia and tubular dysfunction. Williams et al. recently proposed a subtyping criterion for unilateral PA (16): Based on postoperative histology, lesions are categorized as classic or non-classic unilateral disease; Solitary nodules exhibiting characteristic adenoma features on CT—even without CYP11B2 immunohistochemical staining—are classified as classic APA. Notably, unilateral PA represents the most common surgically curable hypertension, with AVS remaining the diagnostic gold standard (4). Controversy persists regarding optimal LI thresholds: while most centers use unstimulated LI cutoffs of 2.0-4.0 and Rossi et al. advocate LI≥2 for optimal diagnosis (42), Kobayashi et al. demonstrate comparable validity for LI≥4 without ACTH stimulation (15). Applying this LI≥4 threshold, our study confirmed significantly elevated PAC in APA versus IHA, alongside greater hypokalemia prevalence and exacerbated renal impairment in classic APA subgroups—findings concordant with established evidence.

Furthermore, this study observed no statistically significant correlation between renal impairment and hypertension duration across patient subgroups. Notably, in the primary aldosteronism (PA) patients, neither the duration of hypokalemia nor hypertension duration showed significant associations with renal damage (*P* > 0.05). These negative findings may reflect the early disease stage of the cohort (mean disease duration <5 years), during which clinically evident renal damage and electrolyte disturbances (e.g., hypokalemia) have not yet manifested. Therefore, early diagnostic confirmation and timely therapeutic intervention are critical for mitigating renal functional decline and improving long-term renal outcomes. Particularly during the compensatory hyperfiltration phase, targeted treatment may halt the progression of nephropathy.

In summary, compared to patients with EH, those with PA exhibit more severe renal impairment, particularly in the aldosterone-producing adenoma (APA) subtype, which can be significantly exacerbated by hypokalemia. Furthermore, both hypokalemia and elevated 24-hour urinary potassium excretion serve as important predictive factors for renal impairment in PA patients. The identification and management of PA at early stages are thus imperative to prevent the progression of renal damage and other complications. However, although this study provides valuable insights into the renal impact of primary aldosteronism (PA), its retrospective design precluded serial monitoring of renal injury markers, limiting assessment of pre- to post-treatment changes in renal parameters and the effects of hypokalemia correction on kidney function. Furthermore, due to the unavailability of CYP11B2 immunohistochemical (IHC) staining, we employed an integrated CT-AVS approach for the further subclassification of the unilateral hypersecretion group. The presence of CT-occult microadenomas may lead to under-detection of atypical cases.

These limitations underscore the need for further prospective studies that can offer a more comprehensive understanding of PA’s progression, treatment efficacy, and outcomes. Such studies should aim to longitudinally assess the impact of targeted interventions, such as adrenalectomy or mineralocorticoid receptor antagonists, on renal function and potassium levels in PA patients. By expanding our knowledge and refining management strategies, we can better address the significant burden of renal complications associated with PA and improve the overall prognosis for these patients.

## Data Availability

The original contributions presented in the study are included in the article/supplementary material. Further inquiries can be directed to the corresponding author.
